# Functional Gene-Guided Discovery of Type II Polyketides from Culturable Actinomycetes Associated with Soft Coral *Scleronephthya* sp

**DOI:** 10.1371/journal.pone.0042847

**Published:** 2012-08-07

**Authors:** Wei Sun, Chongsheng Peng, Yunyu Zhao, Zhiyong Li

**Affiliations:** 1 Marine Biotechnology Laboratory, State Key Laboratory of Microbial Metabolism & School of Life Sciences and Biotechnology, Shanghai Jiao Tong University, Shanghai, People's Republic of China; 2 School of Pharmacy, Shanghai Jiao Tong University, Shanghai, People's Republic of China; Auburn University, United States of America

## Abstract

Compared with the actinomycetes in stone corals, the phylogenetic diversity of soft coral-associated culturable actinomycetes is essentially unexplored. Meanwhile, the knowledge of the natural products from coral-associated actinomycetes is very limited. In this study, thirty-two strains were isolated from the tissue of the soft coral *Scleronephthya* sp. in the East China Sea, which were grouped into eight genera by 16S rDNA phylogenetic analysis: *Micromonospora*, *Gordonia*, *Mycobacterium*, *Nocardioides*, *Streptomyces*, *Cellulomonas*, *Dietzia* and *Rhodococcus*. 6 *Micromonospora* strains and 4 *Streptomyces* strains were found to be with the potential for producing aromatic polyketides based on the analysis of KS_α_ (ketoacyl-synthase) gene in the PKS II (type II polyketides synthase) gene cluster. Among the 6 *Micromonospora* strains, angucycline cyclase gene was amplified in 2 strains (A5-1 and A6-2), suggesting their potential in synthesizing angucyclines *e.g.* jadomycin. Under the guidance of functional gene prediction, one jadomycin B analogue (7b, 13-dihydro-7-O-methyl jadomycin B) was detected in the fermentation broth of *Micromonospora* sp. strain A5-1. This study highlights the phylogenetically diverse culturable actinomycetes associated with the tissue of soft coral *Scleronephthya* sp. and the potential of coral-derived actinomycetes especially *Micromonospora* in producing aromatic polyketides.

## Introduction

Corals are considered as the rainforests of the oceans. Coral-derived natural products span a wide range of chemical classes (*e.g.* prostaglandins, diterpenes, alkaloids and steroids) [1] and display a variety of biological activities (*e.g.* antitumor, anti-inflammatory and antibacterial activities) [2], [3], [4], [5]. Actinomycetes are widely distributed in marine habitats including the sea surface, water column, marine snow, sediments and marine organisms [6], [7], [8], [9], [10], [11], [12]. Excitingly, many previously unknown actinomycete taxa have been successfully isolated from marine habitats [7], [13], [14], [15]. Meanwhile, novel and unique natural products have been increasingly recovered from marine actinomycetes [16], [17], [18], [19], [20]. It has been demonstrated that some compounds originally isolated from marine invertebrates are in fact produced by microorganisms associated with invertebrates [21]. Actinomycetes are frequent components of symbiotic communities in invertebrates [6]. Since coral-associated actinomycetes could play important role in protecting coral host [22], the actinomycetes associated with corals may be involved in the synthesis of natural products isolated from corals. Investigating the coral-associated actinomycetes facilitates to reveal the true origin of biologically active substances, and therefore, is significant for solving the supply problem in marine drug development. However, to date, related reports on coral-associated actinomycetes are still scarce and mainly limited to stony corals [23], [24], [25]. Novel compounds with biological activity have been extracted from soft corals [2], [3], [4], [5], so, it is significant to investigate the soft coral-associated actinomycetes regarding their diversity as well as their potential in secondary metabolite biosynthesis.

Generally, traditional activity-based screening of microbial strains and valuable natural products has its inherent limitation because some natural products cannot be synthesized under the normal condition or the compound yield is very low. With the increasing knowledge of biosynthesis gene cluster for synthesizing natural products, functional gene-based analysis provides a useful approach for predicting natural products [26]. Gene-based analysis has been previously applied in predicting type I polyketide biosynthesis in marine *Actinobacteria* [27]. However, type II polyketide biosynthesis has been rarely concerned. Aromatic polyketides, which are synthesized by type II polyketide synthase (PKS), exhibit a wide array of biological activities including antibacterial, antitumor, antiviral and enzyme inhibitory activities [28], and afford some of the most common antibiotics and anti-cancer drugs currently in clinical use, *e.g.* tetracyclines and anthracyclines. Type II PKS consists of three or more enzymes that act in an iterative manner. The core module in all type II PKS gene clusters is composed of ketoacyl-synthase (KS_α_), chain length factor (KS_β_) and acyl carrier protein (ACP). Moreover, cyclase is responsible for the cyclization of aromatic polyketides. Thus, KS_α_ and cyclase gene can be used as makers for the screening of type II polyketide compounds.

With the aim to reveal the diversity of culturable *Actinobacteria* associated with soft coral and screen the actinomycetes with the potential for synthesizing type II polyketides, actinomycetes were isolated from the tissue of soft coral *Scleronephthya* sp. in the East China Sea. The isolates were tested for their potential in producing aromatic polyketides by the detection of KS_α_ and cyclase gene. Finally, type II polyketide compound was identified in the fermentation broth of *Micromonospora* sp. strain A5-1 under the guidance of functional gene prediction.

## Methods

Ethics Statement: N/A

This study was approved by Shanghai Jiao Tong University, China.

### Sample collection and isolation of actinomycetes

Soft coral *Scleronephthya* sp. was collected from Zhao'an Bay (23°53′N; 117°10′E) in the East China Sea. The sample was stored at −20°C until analysis. Coral tissue was rinsed three times with sterile artificial seawater (ASW) (1.1 g CaCl_2_, 10.2 g MgCl_2_·6H_2_O, 31.6 g NaCl, 0.75 g KCl, 1.0 g Na_2_SO_4_, 2.4 g Tris-HCl, 0.02 g NaHCO_3_, 1L distilled water, pH 7.6) to remove the microbes loosely attached on the surface, and then aseptically grinded using a pestle and a mortar. Six types of media were used for isolating coral-associated actinomycetes [7], [10], [12], [29] (Table S1). All media were supplemented with K_2_Cr_2_O_7_ (50 µg ml^−1^) to inhibit the growth of fungi, and with nalidixic acid (15 µg ml^−1^) to inhibit fast-growing Gram-negative bacteria. Actinomycetes were isolated by serial dilution on agar plates in triplicate at 28°C for 3–6 weeks. The colonies bearing distinct morphological characteristics were picked up and transferred onto freshly prepared plates until pure cultures were obtained.

### Genomic DNA extraction

A single colony was transferred to a 5-ml microtube with 1 ml of liquid medium from which the isolate was originally picked up. The cultures were incubated for 3–5 days at 28°C with shaking at 180 rpm. Microbial cells were collected by centrifugation and genomic DNA was extracted as described by Li and De Boer [30].

### PCR amplification of 16S rRNA gene

The universal bacterial primers 27F (5′-GAGTTTGATCCTGGCTCAG-3′) and 1500R (5′-AGAAAGGAGGTGATCCAGCC-3′) were used for the amplification of 16S rRNA gene [31]. The PCR was carried out in a 20 µl PCR mixtures including 10 µl Taq Premix (Takara, Dalian, China), 0.5 µl 27F (10 µM), 0.5 µl 1500R (10 µM) and 5% DMSO. Cycling conditions were as follows: initial denaturation at 95°C for 3 min, 30 cycles of 94°C for 30 s, 54°C for 40 s, and 72°C for 2 min, and a final extension of 10 min at 72°C.

### PCR amplification of KS_α_ and angucycline cyclase gene

The degenerate primers IIPF6 (5′-TSGCSTGCTTCGAYGCSATC-3′) and IIPR6 (5′-TGGAANCCGCCGAABCCGCT-3′) were employed to amplify the KS_α_ gene of PKS II [32]. The PCR was performed in a 20 µl PCR mixtures including 10 µl Taq Premix, 0.8 µl IIPF6 (25 µM), 0.8 µl IIPR6 (25 µM) and 5% DMSO. Cycling conditions were as follows: initial denaturation at 95°C for 5 min, 30 cycles of 95°C for 35 s, 55°C for 40 s, and 72°C for 1 min, and a final extension of 10 min at 72°C. The degenerate primers AuF3 (5′-GAACTGGCCSCGSRTBTT-3′) and AuR4 (5′-CCNGTGTGSARSKTCATSA-3′) were applied in the amplification of angucycline cyclase gene [33]. 20 µl PCR mixtures included 10 µl Taq Premix, 1 µl AuF3 (40 µM), 1 µl AuR4 (40 µM) and 5% DMSO. Cycling conditions were as follows: initial denaturation at 94°C for 5 min, 30 cycles of 94°C for 45 s, 60°C for 1 min, and 72°C for 1 min, and a final extension of 8 min at 72°C.

### Sequencing and phylogenetic analyses

The PCR products were purified using Agarose Gel DNA Purification Kit (Takara, Dalian, China) and sequenced on an ABI 3730 automated sequencer by Beijing Genomic Institute (Shenzhen, China). The gene sequences obtained were proofread using Chromas, version 1.62 (Technelysium). The nucleotide sequences were matched with published sequences in NCBI using the BLAST search program (http://www.ncbi.nlm.nih.gov/). For KS_α_ and cyclase gene, translated protein sequences were derived from nucleotide sequences using the ORF FINDER available at the NCBI (http://www.ncbi.nlm.nih.gov/projects/gorf/). The deduced amino acid sequences were used as queries to search the related proteins in the nr protein database using the BLASTP algorithm. For 16S rRNA gene and KS_α_, multiple sequence alignment was performed using CLUSTALX. Phylogenetic tree was constructed using Mega 4 [34]. The consistency of the trees was verified by bootstrapping (1,000 replicates) for parsimony.

### Nucleotide sequence accession numbers

16S rRNA, KS_α_ (PKS II) and angucycline cyclase gene sequences from the soft coral-derived actinomycete isolates were deposited in the GenBank database under the following accession numbers: JN627163–JN627194, JN627195–JN627204 and JQ943912–JQ943913.

### Fermentation and chemical identification

Strain A5-1 was inoculated in 25 ml flask using GYMM medium (20 g glycerol, 10 g yeast extract, 4 g malt extract, 10 g mannitol, 1 liter ASW) at 28°C,180 rpm in the dark for 3 weeks, and then transferred to a 250 ml Erlenmeyer flask containing 100 ml of D-galactose-L-isoleucine medium [35]. The culture was incubated at 28°C, 180 rpm in the dark for 45 days. On the fifteenth day, ethanol was added to a final concentration of 6% (v/v) to induce the synthesis of jadomycin [35].

After mycelium was removed by filtration, the fermentation broth was extracted with 100 ml of acetic ether (EtOAc) and concentrated in vacuo. EtOAc extract was dissolved in methanol for HPLC-DAD analysis on an Agilent 1200 (Agilent Technologies, USA) series with an on-line Diode Array Detector (DAD/UV) and a C_18_ RP-column (Eclipse XDB-C_18_ 5 µm, 4.6×150 mm). Ultraviolet absorption was compared with that of jadomycins according to their maximum absorption wavelength (λ_max_) [36].

For LC-QTOF-MS analysis, the methanol solution of strain A5-1 extract was detected on an ultra performance liquid & quadrupole time of flight mass spectroscopy (UPLC-QTOF-MS Premier, Waters Corporation, USA). The analytes were separated on a C_18_ RP-column (ACQUITY BEH-C_18_ 1.7 µm, 2.1×100 mm, Waters Co.), with linear gradient elution from H_2_O (1‰ formic acid) to 35% H_2_O/MeCN (1‰ formic acid). Total ions chromatography (TIC) and mass spectrum of selected ion were acquired in positive electro-spray ionization mass spectrum (ESI-MS) mode.

In the case of ^1^H NMR analysis, the EtOAc extract was dried in vacuo and then dissolved in CD_3_OD. Proton nuclear magnetic resonance (^1^H NMR) spectrum was recorded on an AVANCE III 400 spectrometer (400 MHz, Bruker).

## Results

### Recovery and phylogenetic diversity of coral *Scleronephthya* sp.-associated actinomycetes

**Figure 1:**
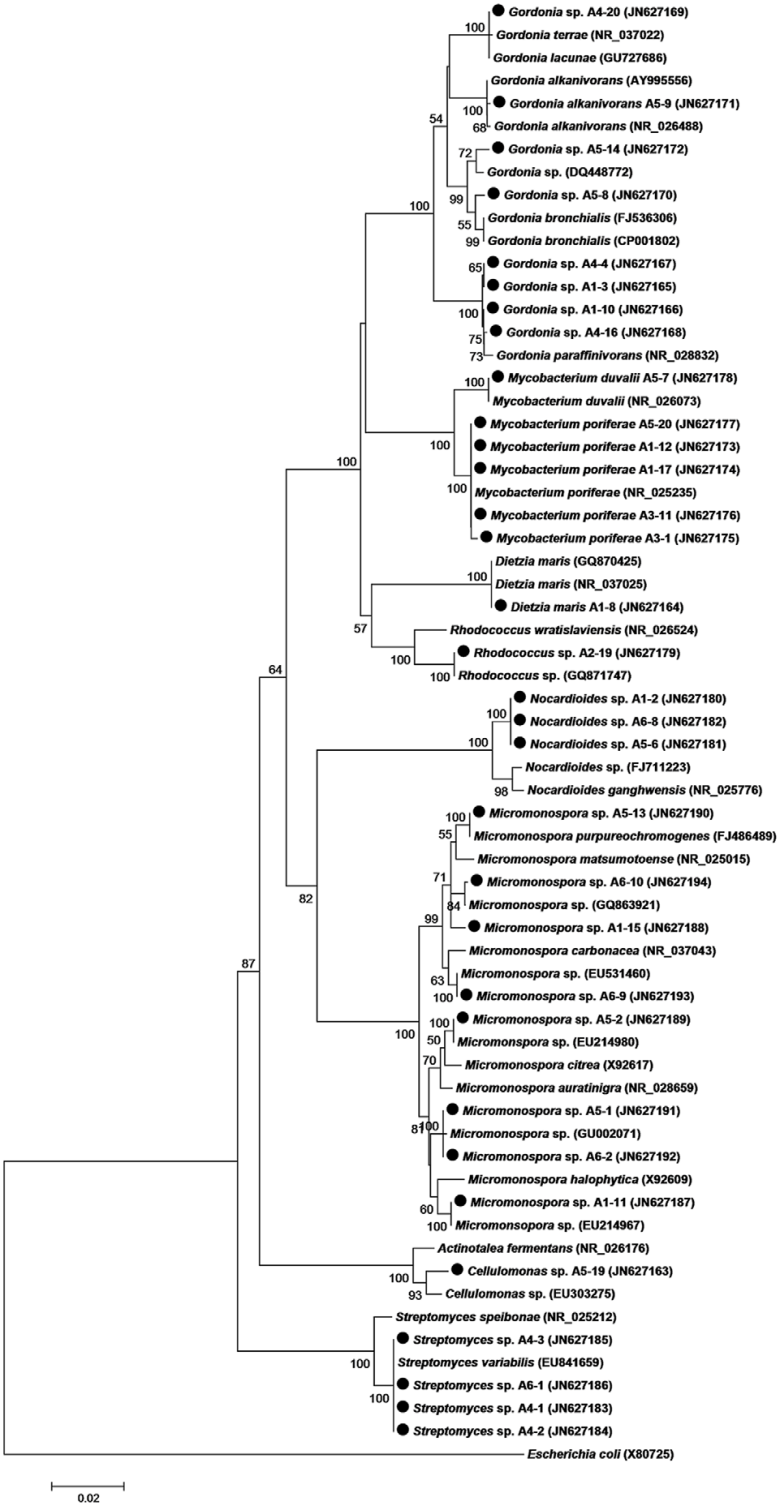
Neighbor-joining phylogenetic tree based on 16S rRNA gene sequence (*ca*.1,400 bp) of actinomycetes from the tissue of soft coral *Scleronephthya* sp. The sequences obtained in this work are marked by black dot. The number is the percentage indicating the level of boot strap support, based on a neighbor-joining analysis of 1,000 resampled data sets. The scale bar represents 0.02 substitutions per nucleotide position.

After incubation for 6 weeks, 32 isolates were recovered. Based on the BLAST analyses of 16S rRNA gene sequences, these 32 isolates were assigned to *Actinobacteria* with 98–100% similarity to their nearest relatives in the GenBank database, including 8 genera: *Micromonospora* (8 isolates), *Gordonia* (8 isolates), *Mycobacterium* (6 isolates), *Nocardioides* (3 isolates), *Streptomyces* (4 isolates), *Cellulomonas* (1 isolate), *Dietzia* (1 isolate) and *Rhodococcus* (1 isolate) (Table 1; Figure 1), which indicated that *Micromonospora* and *Gordonia* are relatively dominant among the culturable actinomycetel community in the tissue of the soft coral *Scleronephthy*a sp.. Four strains (*Gordonia* sp. strain A5-14, *Rhodococcus* sp. strain A2-19, *Micromonospora* sp. strains A1-11 and A5-2) share high homology with relatives derived from marine sediments. Eight strains (*Mycobacterium poriferae* strains A1-12, A1-17, A3-1, A3-11 and A5-20, *Micromonospora* sp. strains A5-1, A6-2 and A6-10) show high similarity to relatives isolated from marine sponges.

**Table 1:** Actinomycetes and those with PKS II gene from the soft coral *Scleronephthya* sp.

Genus	Strain (NCBI accession no.)	Most closely related strain (NCBI accession no.)	Identity (%)	PKS II
*Cellulomonas*	A5-19 (JN627163)	*Cellulomonas* sp.(EU303275)	98	−
*Dietzia*	A1-8 (JN627164)	*D. maris* (GQ870425)	99	−
*Gordonia*	A1-3 (JN627165)	*G. paraffinivorans* (NR_028832)	99	−
	A1-10 (JN627166)	*G. paraffinivorans* (NR_028832)	99	−
	A4-4 (JN627167)	*G. paraffinivorans* (NR_028832)	99	−
	A4-16 (JN627168)	*G. paraffinivorans* (NR_028832)	99	−
	A4-20 (JN627169)	*G. lacunae* (GU727686)	99	−
	A5-8 (JN627170)	*G. bronchialis* (FJ536306)	99	−
	A5-9 (JN627171)	*G. alkanivorans* (AY995556)	99	−
	A5-14 (JN627172)	*Gordonia* sp. (DQ448772)	99	−
*Mycobacterium*	A1-12 (JN627173)	*M. poriferae* (NR_025235)	99	−
	A1-17 (JN627174)	*M. poriferae* (NR_025235)	99	−
	A3-1 (JN627175)	*M. poriferae* (NR_025235)	99	−
	A3-11 (JN627176)	*M. poriferae* (NR_025235)	99	−
	A5-20 (JN627177)	*M. poriferae* (NR_025235)	99	−
	A5-7 (JN627178)	*M. duvalii* (NR_026073)	100	−
*Rhodococcus*	A2-19 (JN627179)	*Rhodococcus* sp. (GQ871747)	99	−
*Nocardioides*	A1-2 (JN627180)	*Nocardioides* sp. (FJ711223)	99	−
	A5-6 (JN627181)	*Nocardioides* sp. (FJ711223)	98	−
	A6-8 (JN627182)	*Nocardioides* sp. (FJ711223)	99	−
*Streptomyces*	A4-1 (JN627183)	*S. variabilis* (EU841659)	99	+
	A4-2 (JN627184)	*S. variabilis* (EU841659)	100	+
	A4-3 (JN627185)	*S. variabilis* (EU841659)	99	+
	A6-1 (JN627186)	*S. variabilis* (EU841659)	99	+
*Micromonospora*	A1-11 (JN627187)	*Micromonospora* sp. (EU214967)	99	−
	A1-15 (JN627188)	*Micromonospora* sp. (EU531460)	99	+
	A5-2 (JN627189)	*Micromonospora* sp. (EU214980)	99	+
	A5-13 (JN627190)	*M. purpureochromogenes* (FJ486489)	100	+
	A5-1 (JN627191)	*Micromonospora* sp. (GU002071)	99	+
	A6-2 (JN627192)	*Micromonospora* sp. (GU002071)	99	+
	A6-9 (JN627193)	*Micromonospora* sp. (EU531460)	100	+
	A6-10 (JN627194)	*Micromonospora* sp. (GQ863921)	99	−

Notably, significant differences in the total number of isolates were observed among the 6 different media (Figure S1). M5 produced the highest recovery with 10 isolates, followed by M1 (8 isolates), M4 (6 isolates), M6 (5 isolates), M3 (2 isolates) and M2 (1 isolate). Additionally, the actinomycete diversity recovered from the different media varied (Figure S1). For example, M1 and M5 yielded the highest diversity with 5 genera, followed by M6 (3 genera), M4 (2 genera), M2 (1 genus) and M3 (1 genus). As expected, the combination of 6 media achieved a better recoverability of coral-associated actinomycetes.

### The potential for producing type II polyketides based on functional gene analysis

The presence of KS_α_ gene was detected in two of the eight genera, *Streptomyces* (4 strains) and *Micromonospora* (6 strains) (Table 1). Based on BLAST analyses, the KS_α_ sequences from four *Streptomyces* strains show high (98.4–98.8%) sequence similarity to their BLAST matches, whereas, the KS_α_ sequences from six *Micromonospora* strains share relatively lower (<89.4%) homology with previously reported sequences.

**Figure 2:**
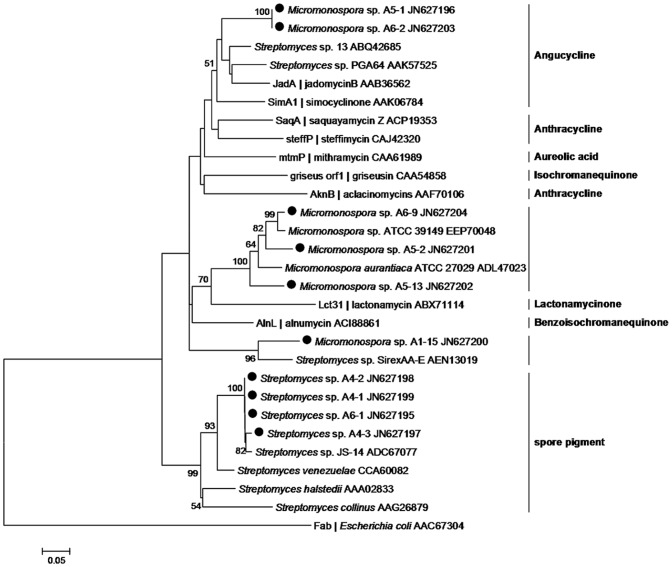
Neighbor-joining tree constructed using aligned KS_α_ domain amino acid sequence (203 amino acid positions) from type II PKSs. The sequences obtained in this work are marked by black dot. Next to the taxon name, GenBank accession number of KS_α_ domain amino acid sequence or/and the identified compounds are indicated. Bootstrap values calculated from 1,000 resamplings using neighborjoining are shown at the respective nodes when the calculated values were 50% or greater. The scale bar represents 0.05 substitutions per amino acid position.

A phylogenetic tree was generated using 10 KS_α_ amino acid sequences obtained in this study and 17 reference sequences retrieved from GenBank (Figure 2). Reference sequences related to biosynthetic pathways help to group the obtained sequences into different clusters representing different chemotypes. As shown in Fig. 2, KS_α_ sequences from 6 *Micromonospora* strains are separated into three major phylogenetic divisions. For example, sequences from strains A5-1 and A6-2 fall into a cluster with angucycline ketosynthase sequences, and show the closest evolutionary relationship with *Jad A* (AAB36562) which is involved in the biosynthesis of jadomycin B (Table 2). Sequences of strains A5-2, A6-9 and A5-13 are clustered in a group together with relative *Lac 31* (ABX71114) associated with the biosynthetic pathway of lactonamycin. Interestingly, the unique KS_α_ sequence from strain A1-15 is clearly separated from any known sequence involved in characterized pathways. After the phylogenetic analysis, 32 strains were tested for the presence of angucycline cyclase gene which is involved in the aromatization of angucycline. The target band of approximately 650 bp was successfully amplified in *Micromonospora* sp. strains A5-1 and A6-2. This result indicates that these two *Micromonospora* strains have the potential in producing angucycline compounds such as jadomycin.

**Table 2:** KS_α_ nucleotide sequences.

Strain	NCBI accession no.	Top BLAST match (NCBI accession no.)	Identity (%)
*Micromonospora* sp. A1-15	JN627200	*Streptomyces* sp. SirexAA-E β-ketoacyl synthase gene (CP002993)	85.8
*Micromonospora* sp. A5-1	JN627196	*Micromonospora* sp. SAUK6030 type II polyketide synthase-like gene (GQ118939)	86.9
		*Streptomyces venezuelae* jadomycin polyketide ketosynthase (jadA) gene (AF126429)	85.8
*Micromonospora* sp. A5-2	JN627201	*Micromonospora aurantiaca* ATCC 27029 β-ketoacyl synthase gene (CP002162)	88.2
*Micromonospora* sp. A5-13	JN627202	*Micromonospora aurantiaca* ATCC 27029 β-ketoacyl synthase gene (CP002162)	88.9
*Micromonospora* sp. A6-2	JN627203	*Micromonospora* sp. SAUK6030 type II polyketide synthase-like gene (GQ118939)	86.7
		*Streptomyces venezuelae* jadomycin polyketide ketosynthase (jadA) gene (AF126429)	85.8
*Micromonospora* sp. A6-9	JN627204	*Micromonospora aurantiaca* ATCC 27029 β-ketoacyl synthase gene (CP002162)	89.4
*Streptomyces* sp. A4-1	JN627199	*Streptomyces* sp. JS-14 ketosynthase gene (GU373728)	98.7
*Streptomyces* sp. A4-2	JN627198	*Streptomyces* sp. JS-14 ketosynthase gene (GU373728)	98.8
*Streptomyces* sp. A4-3	JN627197	*Streptomyces* sp. JS-14 ketosynthase gene (GU373728)	98.5
*Streptomyces* sp. A6-1	JN627195	*Streptomyces* sp. JS-14 ketosynthase gene (GU373728)	98.4

### The identification of a novel analogue of jadomycin B in the fermentation broth of *Micromonospora* sp. strain A5-1

Among *Micromonospora* sp. strains A5-1 and A6-2 with potential to produce jadomycin B or its analogues, strain A5-1 was selected for fermentation to test the gene prediction since the two strains belong to the same species. Only 10 mg EtOAc extract of the fermentation broth of *Micromonospora* sp. strain A5-1 was obtained because *Micromonospora* sp. strain A5-1 grew very slowly and the biomass was very low.

**Figure 3:**
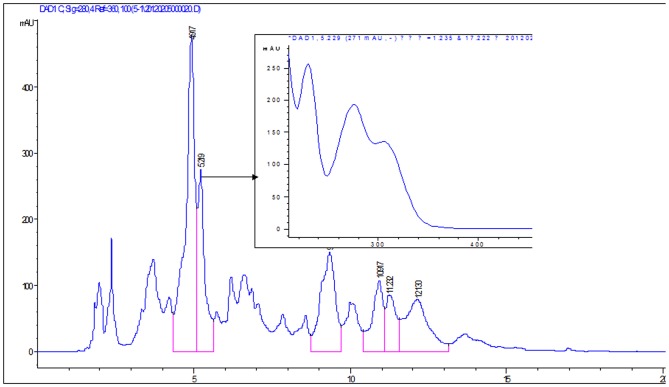
HPLC of the EtOAc extract of *Micromonospora* sp. strain A5-1 fermentation broth (UV spectra of selected peaks at t_R_ 5.22 min show similar absorption as jadomycins).

Jadomycin B displays 5 UV absorptions: 212 nm, 238 nm, 280 nm, 312 nm and 520 nm [36]. In the EtOAc extract of fermentation broth of *Micromonospora* sp. strain A5-1, one peak (retention time (t_R_) at 5.22 min, Figure 3) shows similar UV profiles as that of jadomycins except the absorption band over 350 nm which is contributed by the substructure of *p*-quinone. The result suggests the existence of jadomycin B analogue with one keto function reduction in the fermentation broth of *Micromonospora* sp. strain A5-1.

**Figure 4:**
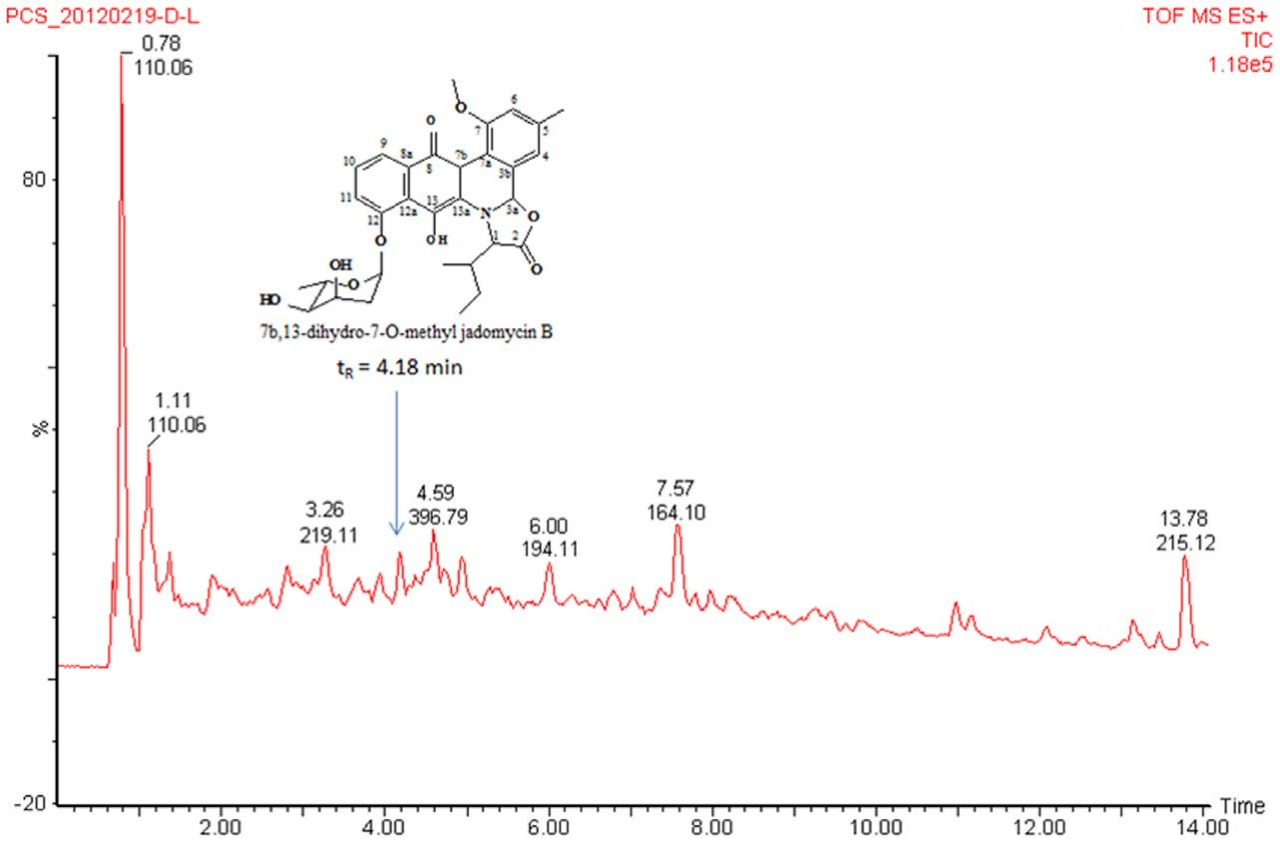
TIC of the EtOAc extract of *Micromonospora* sp. strain A5-1 fermentation broth (the peak with t_R_ at 4.18 min is putative jadomycin analogue).

**Figure 5:**
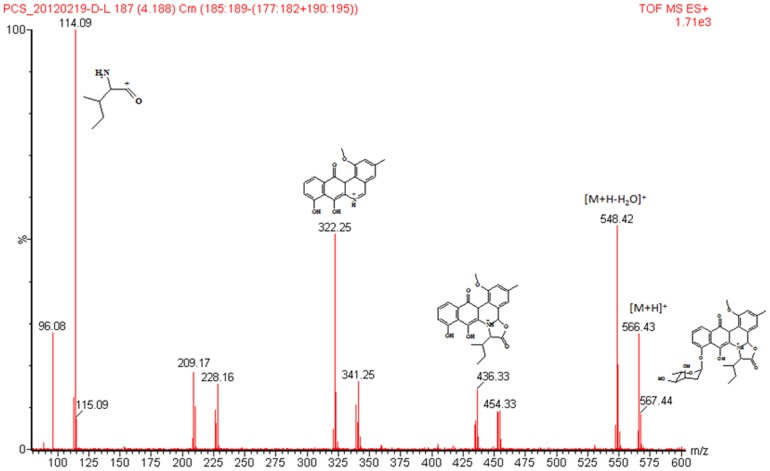
Mass spectrum of selected ion at t_R_ 4.18 min in TIC.

**Figure 6:**
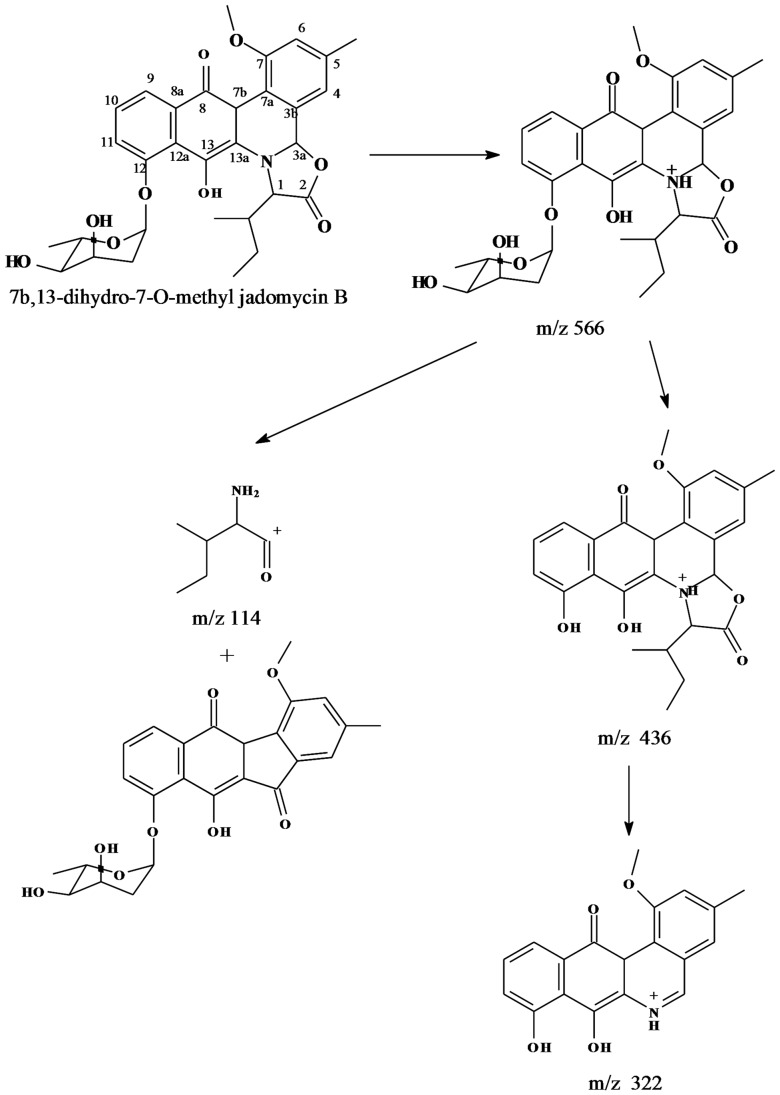
Suggested fragmentation process of selected ion at t_R_ 4.18 min in TIC.

**Figure 7:**
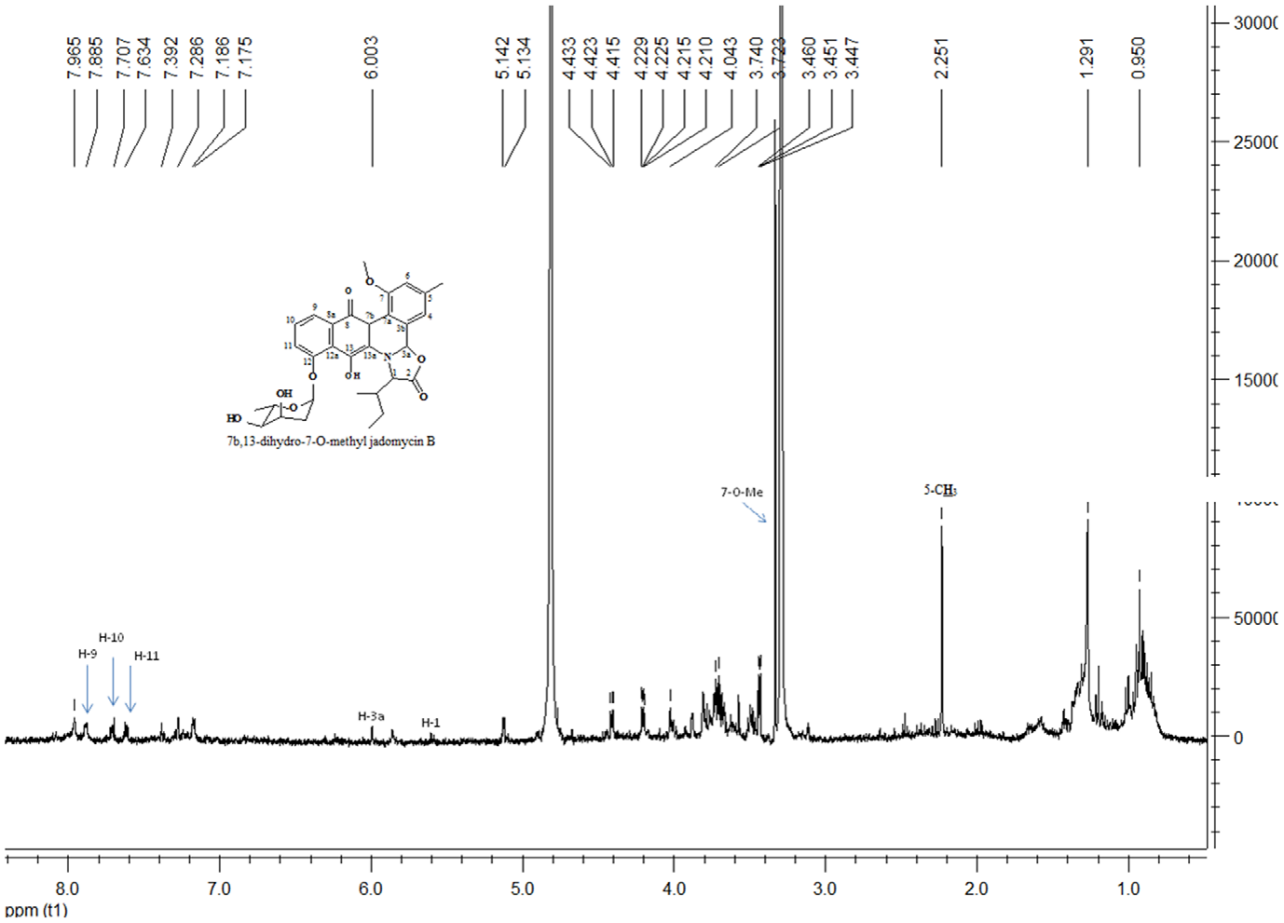
^1^H NMR data of selected ion at t_R_ 4.18 min in TIC.

Jadomycin B shows mass to charge (m/z) at 306 and 550 in ESI mass spectrum which are assigned as key fragmentation ion [phenanthroviridin+H]^+^ and pseudomolecular ion [jadomycin B+H]^+^ [37]. In this study, TIC of the EtOAc extract of *Micromonospora* sp. strain A5-1 fermentation broth shows one m/z 566 with t_R_ at 4.18 min (Figure 4), which is 16 amu more than that of pseudo-molecular ion of jadomycin B. So, the 14 amu corresponding to methylene should be added to the keto reduction derivative of jadomycin B. In the mass spectrum (Figure 5), the key fragmentation ion at m/z 322 instead of that at m/z 306 of jadomycins supports the change in phenanthroviridin. Based on the spectral data analysis and comparison with jadomycin B, the putative structure of target compound corresponding to the peak with t_R_ at 4.18 min in Figure 4 should be 7b, 13-dihydro-7-O-methyl jadomycin B. The possible MS fragmentations are shown in Figures 5 & 6. Meanwhile, this assignment is also supported by the ^1^H NMR data (Figure 7), which are consistent with that of jadomycin B [36].

## Discussion

### The phylogenetic diversity of culturable actinomycetes associated with coral *Scleronephthya* sp

Studies on sponge-associated actinomycetes indicate that medium exhibits significant effect on the diversity of *Actinobacteria* recovered [12], [38]. So, in order to gain a better recoverability of coral-associated actinomycetes, six different media were used in this study. Similarly, medium-dependent recovery efficiency was observed. Taking the dominant *Micromonospora* for example, it was recovered from only 3 types of media. Moreover, not any one medium can recover all 8 genera, suggesting the necessity of combining different media to increase the recovery rate of cultured actinomycetes.

Prior to this study, the investigation of culturable actinomycetes has been mainly focused on stony corals [23], [24], [25]. In this study, a total of 8 genera were successfully isolated from the soft coral *Scleronephthya* sp., including *Micromonospora*, *Gordonia*, *Mycobacterium*, *Nocardioides*, *Streptomyces*, *Cellulomonas*, *Dietzia* and *Rhodococcus*. The culturable actinomycetes include both common and rare actinomycetes species. Rare actinomycetes derived from marine habitats, such as *Salinispora* [39], *Verrucosispora* [40] and *Micromonospora* [41], [42], [43], have shown their unique capacity to produce novel natural products. BLAST analyses shows that the isolated actinomycete strains *e.g. Micromonospora*, *Mycobacterium*, *Gordonia* and *Rhodococcus* have closest relatives derived from marine sponges or marine sediments. *Mycobacterium poriferae* was originally isolated from the sponge *Halichondria bowerbanki* [44]. Recently, 11 strains of *M. poriferae* have been isolated from the sponge *Amphimedon queenslandica* and the authors proposed that the isolates may represent a sponge-specific phylotype [45]. It is worth noting that, in this study, 5 strains *M. poriferae* were isolated from the tissue of this soft coral, suggesting that *M. poriferae* are not merely limited in sponges.

### The potential of culturable actinomycetes associated with coral *Scleronephthya* sp. in producing type II polyketides

It is proposed that actinomycetes with PKS gene do produce a larger number of new metabolites [26]. In this study, actinomycetes with the potential to produce aromatic polyketides were screened by detecting KS_α_ and cyclase genes of PKS II. Among the 32 strains actinomycetes, 10 strains from two genera *Streptomyces* and *Micromonospora* yielded positive results. *Streptomyces* is a well-known polyketide producer, so it is not surprising that KS_α_ gene was identified in all the 4 *Streptomyces* strains. Prior to this study, it was found that most of the *Micromonospora* strains are not potential producers of type II polyketides [26], [33]. The known secondary metabolites produced by *Micromonospora* are mainly aminoglycosides, macrolides and enediynes, few aromatic polyketides are known to be produced by *Micromonospora* except anthracyclines [46]. In contrast, it is unexpected that the target gene was detected in 6 of 8 *Micromonospora* strains, indicating that some coral-associated *Micromonospora* strains have the potential in producing aromatic polyketides.

Early in 1994, it was known that the production of jadomycin B in *Streptomyces venezuelae* ISP5230 needed to be induced by heat shock, ethanol treatment or phage infection [35]. Apparently, the jadomycin pathway is cryptic and only activated under specific conditions. In this case, natural product discovery strategy based on traditional bioassay is limited. Similarly, the D-galactose-L-isoleucine medium, which is beneficial for producing jadomycin B [35], was used in the fermentation of *Micromonospora* sp. strain A5-1, followed with ethanol induction [35]. Although jadomycin B was not found in the fermentation broth of *Micromonospora* sp. strain A5-1, a novel analogue of jadomycin B, *i.e.* 7b, 13-dihydro-7-O-methyl jadomycin B, was identified, which proved the prediction based on the functional gene screening. This study indicates that gene-based screening may guide the discovery of target metabolites especially those cannot be synthesized under the normal cultivation conditions. However, because *Micromonospora* sp. strain A5-1 grew very slowly and the yield of target compound was very low, so, in this study, the pure 7b, 13-dihydro-7-O-methyl jadomycin B was not isolated successfully. Alternatively, for the slowly-growing *Micromonospora* with type II polyketides producing potential, the cloning and heterologous expression of related gene cluster is a potential choice for future investigation.

The results from this study indicate that the soft coral tissue harbors diverse actinomycetes, some of which are with potential in synthesizing type II polyketides. This study, together with actinomycetes from stony corals [23], [24], [25], [47], suggests that the diverse culturable coral-associated actinomycetes are important source for marine natural products.

## Supporting Information

Table S1 **Media used for the isolation of actinomycetes from the soft coral** ***Scleronephthya*** **sp.**
(DOC)

Figure S1 **The diversity of actinomycetes recovered using six media.**
(DOC)

## References

[1] Blunt JW, Copp BR, Hu WP, Munro MHG, Northcote PT, et al (2009) Marine natural products. Nat Prod Rep 26: 170–244. 19177222 10.1039/b805113p

[2] Yan XH, Gavagnin M, Cimino G, Guo YW (2007) Two new biscembranes with unprecedented carbon skeleton and their probable biogenetic precursor from the Hainan soft coral *Sarcophyton latum* . Tetrahedron Lett 48: 5313–5316.

[3] Han L, Wang CY, Huang H, Shao CL, Liu QA, et al (2010) A new pregnane analogue from Hainan soft coral *Scleronephthya gracillimum* Kükenthal. Biochem Syst Ecol 38: 243–246.

[4] Li L, Sheng L, Wang CY, Zhou YB, Huang H, et al (2011) Diterpenes from the Hainan soft coral *Lobophytum cristatum* Tixier-Durivault. J Nat Prod 74: 2089–2094. 21954851 10.1021/np2003325

[5] Yan XH, Liu HL, Huang H, Li XB, Guo YW (2011) Steroids with aromatic A-rings from the Hainan soft coral *Dendronephthya studeri* Ridley. J Nat Prod 74: 175–180. 21192715 10.1021/np100562n

[6] Ward AC, Bora N (2006) Diversity and biogeography of marine actinobacteria. Curr Opin Microbiol 9: 279–286. 16675292 10.1016/j.mib.2006.04.004

[7] Mincer TJ, Jensen PR, Kauffman CA, Fenical W (2002) Widespread and persistent populations of a major new marine actinomycete taxon in ocean sediments. Appl Environ Microb 68: 5005–5011. 10.1128/AEM.68.10.5005-5011.2002 PMC126404 12324350

[8] Bredholdt H, Galatenko OA, Engelhardt K, Fjaervik E, Terekhova LP, et al (2007) Rare actinomycete bacteria from the shallow water sediments of the Trondheim fjord, Norway: isolation, diversity and biological activity. Environ Microbiol 9: 2756–2764. 17922759 10.1111/j.1462-2920.2007.01387.x

[9] Anzai K, Nakashima T, Kuwahara N, Suzuki R, Ohfuku Y, et al (2008) Actinomycete bacteria isolated from the sediments at coastal and offshore area of nagasaki prefecture, Japan: diversity and biological activity. J Biosci Bioeng 106: 215–217. 18804069 10.1263/jbb.106.215

[10] Zhang HT, Lee YK, Zhang W, Lee HK (2006) Culturable actinobacteria from the marine sponge *Hymeniacidon perleve*: isolation and phylogenetic diversity by 16S rRNA gene-RFLP analysis. Antonie van Leeuwenhoek 90: 159–169. 16871424 10.1007/s10482-006-9070-1

[11] Jiang SM, Sun W, Chen MJ, Dai SK, Zhang L, et al (2007) Diversity of culturable actinobacteria isolated from marine sponge *Haliclona* sp.. Antonie van Leeuwenhoek 92: 405–416. 17566868 10.1007/s10482-007-9169-z

[12] Abdelmohsen UR, Pimentel-Elardo SM, Hanora A, Radwan M, Abou-El-Ela SH, et al (2010) Isolation, phylogenetic analysis and anti-infective activity screening of marine sponge-associated actinomycetes. Mar Drugs 8: 399–412. 20411105 10.3390/md8030399 PMC2857355

[13] Zhang HT, Zheng W, Huang JY, Luo HL, Jin Y, et al (2006) *Actinoalloteichus hymeniacidonis* sp. nov., an actinomycete isolated from the marine sponge *Hymeniacidon perleve* . Int J Syst Evol Micr 56: 2309–2312. 10.1099/ijs.0.64217-0 17012552

[14] Olson JB, Harmody DK, Bej AK, McCarthy PJ (2007) *Tsukamurella spongiae* sp. nov., a novel actinomycete isolated from a deep-water marine sponge. Int J Syst Evol Micr 57: 1478–1481. 10.1099/ijs.0.64837-0 17625179

[15] Xiao J, Luo YX, Xie SJ, Xu J (2011) *Serinicoccus profundi* sp. nov., an actinomycete isolated from deep-sea sediment, and emended description of the genus *Serinicoccus* . Int J Syst Evol Micr 61: 16–19. 10.1099/ijs.0.019976-0 20118285

[16] Bull AT, Stach JEM (2007) Marine actinobacteria: new opportunities for natural product search and discovery. Trends Microbiol 15: 491–499. 17997312 10.1016/j.tim.2007.10.004

[17] Macherla VR, Liu J, Sunga M, White DJ, Grodberg J, et al (2007) Lipoxazolidinones A, B, and C: antibacterial 4-oxazolidinones from a marine actinomycete isolated from a Guam marine sediment. J Nat Prod 70: 1454–1457. 17845000 10.1021/np0702032

[18] Martin GDA, Tan LT, Jensen PR, Dimayuga RE, Fairchild CR, et al (2007) Marmycins A and B, cytotoxic pentacyclic C-glycosides from a marine sediment-derived actinomycete related to the genus *Streptomyces* . J Nat Prod 70: 1406–1409. 17844998 10.1021/np060621r

[19] Khan ST, Komaki H, Motohashi K, Kozone I, Mukai A, et al (2011) *Streptomyces* associated with a marine sponge *Haliclona* sp.; biosynthetic genes for secondary metabolites and products. Environ Microbiol 13: 391–403. 20849448 10.1111/j.1462-2920.2010.02337.x

[20] Li K, Li QL, Ji NY, Liu B, Zhang W, et al (2011) Deoxyuridines from the marine sponge associated actinomycete *Streptomyces microflavus* . Mar Drugs 9: 690–695. 21673882 10.3390/md9050690 PMC3111175

[21] Radjasa OK, Vaske YM, Navarro G, Vervoort HC, Tenney K, et al (2011) Highlights of marine invertebrate-derived biosynthetic products: Their biomedical potential and possible production by microbial associants. Bioorg Med Chem 19: 6658–6674. 21835627 10.1016/j.bmc.2011.07.017 PMC3205244

[22] Nithyanand P, Thenmozhi R, Rathna J, Pandian SK (2010) Inhibition of *Streptococcus pyogenes* biofilm formation by coral-associated actinomycetes. Curr Microbiol 60: 454–460. 20020301 10.1007/s00284-009-9564-y

[23] Lampert Y, Kelman D, Dubinsky Z, Nitzan Y, Hill RT (2006) Diversity of culturable bacteria in the mucus of the Red Sea coral *Fungia scutaria* . FEMS Microbiol Ecol 58: 99–108. 16958911 10.1111/j.1574-6941.2006.00136.x

[24] Nithyanand P, Pandian SK (2009) Phylogenetic characterization of culturable bacterial diversity associated with the mucus and tissue of the coral *Acropora digitifera* from the Gulf of Mannar. FEMS Microbiol Ecol 69: 384–394. 19619231 10.1111/j.1574-6941.2009.00723.x

[25] Nithyanand P, Manju S, Pandian SK (2011) Phylogenetic characterization of culturable actinomycetes associated with the mucus of the coral *Acropora digitifera* from Gulf of Mannar. FEMS Microbiol Lett 314: 112–118. 21105906 10.1111/j.1574-6968.2010.02149.x

[26] Schneemann I, Nagel K, Kajahn I, Labes A, Wiese J, et al (2010) Comprehensive investigation of marine actinobacteria associated with the sponge *Halichondria panicea* . Appl Environ Microb 76: 3702–3714. 10.1128/AEM.00780-10 PMC2876447 20382810

[27] Gontang EA, Gaudêncio SP, Fenical W, Jensen PR (2010) Sequence-based analysis of secondary-metabolite biosynthesis in marine actinobacteria. Appl Environ Microb 76: 2487–2499. 10.1128/AEM.02852-09 PMC2849207 20154113

[28] Hertweck C, Luzhetskyy A, Rebets Y, Bechthold A (2007) Type II polyketide synthases: gaining a deeper insight into enzymatic teamwork. Nat Prod Rep 24: 162–190. 17268612 10.1039/b507395m

[29] Webster NS, Wilson KJ, Blackall LL, Hill RT (2001) Phylogenetic diversity of bacteria associated with the marine sponge *Rhopaloeides odorabile* . Appl Environ Microb 67: 434–444. 10.1128/AEM.67.1.434-444.2001 PMC92596 11133476

[30] Li X, De Boer SH (1995) Selection of polymerase chain reaction primers from an RNA intergenic spacer region for specific detection of *Clavibacter michiganensis* subsp. *sepedonicus* . Phytopathol 85: 837–842.

[31] Woese CR, Gutell R, Gupta R, Noller HF (1983) Detailed analysis of the higher-order structure of 16S-like ribosomal ribonucleic acids. Microbiol Rev 47: 621–669. 6363901 10.1128/mr.47.4.621-669.1983 PMC283711

[32] Metsä-Ketelä M, Salo V, Halo L, Hautala A, Hakala J, et al (1999) An efficient approach for screening minimal PKS genes from *Streptomyces* . FEMS Microbiol Lett 180: 1–6. 10547437 10.1111/j.1574-6968.1999.tb08770.x

[33] Ouyang YC, Wu HB, Xie LW, Wang GH, Dai SK, et al (2011) A method to type the potential angucycline producers in actinomycetes isolated from marine sponges. Antonie van Leeuwenhoek 99: 807–815. 21287404 10.1007/s10482-011-9554-5

[34] Tamura K, Dudley J, Nei M, Kumar S (2007) MEGA4: Molecular evolutionary genetics analysis (MEGA) software version 4.0. Mol Biol Evol 24: 1596–1599. 17488738 10.1093/molbev/msm092

[35] Doull JL, Singh AK, Hoare M, Ayer SW (1994) Conditions for the production of jadomycin B by *Streptomyces venezuelae* ISP5230: effects of heat shock, ethanol treatment and phage infection. J Ind Microbiol 13: 120–125. 7764672 10.1007/BF01584109

[36] Rix U, Zheng JT, Rix LLR, Greenwell L, Yang K, et al (2004) The dynamic structure of jadomycin B and the amino acid incorporation step of its biosynthesis. J Am Chem Soc 126: 4496–4497. 15070349 10.1021/ja031724o

[37] Jakeman DL, Farrell S, Young W, Doucet RJ, Timmons SC (2005) Novel jadomycins: incorporation of non-natural and natural amino acids. Bioorg Med Chem Lett 15: 1447–1449. 15713404 10.1016/j.bmcl.2004.12.082

[38] Zhang HT, Zhang W, Jin Y, Jin MF, Yu XJ (2008) A comparative study on the phylogenetic diversity of culturable actinobacteria isolated from five marine sponge species. Antonie van Leeuwenhoek 93: 241–248. 17717723 10.1007/s10482-007-9196-9

[39] Williams PG, Buchanan GO, Feling RH, Kauffman CA, Jensen PR, et al (2005) New cytotoxic salinosporamides from the marine actinomycete *Salinispora tropica* . J Org Chem 70: 6196–6203. 16050677 10.1021/jo050511+

[40] Fiedler HP, Bruntner C, Riedlinger J, Bull AT, Knutsen G, et al (2008) Proximicin A, B and C, novel aminofuran antibiotic and anticancer compounds isolated from marine strains of the actinomycete *Verrucosispora* . J Antibiot 61: 158–163. 18503194 10.1038/ja.2008.125

[41] Romero F, Espliego F, Baz JP, De Quesada TG, Gravalos D, et al (1997) Thiocoraline, a new depsipeptide with antitumor activity produced by a marine *Micromonospora*.1. Taxonomy, fermentation, isolation, and biological activities. J Antibiot 50: 734–737. 9360617 10.7164/antibiotics.50.734

[42] Baz JP, Canedo LM, Puentes JLF, Elipe MVS (1997) Thiocoraline, a novel depsipeptide with antitumor activity produced by a marine *Micromonospora*.2. Physico-chemical properties and structure determination. J Antibiot 50: 738–741. 9360618 10.7164/antibiotics.50.738

[43] Charan RD, Schlingmann G, Janso J, Bernan V, Feng XD, et al (2004) Diazepinomicin, a new antimicrobial alkaloid from a marine *Micromonospora* sp.. J Nat Prod 67: 1431–1433. 15332871 10.1021/np040042r

[44] Padgitt PJ, Moshier SE (1987) *Mycobacterium poriferae* sp. nov., a scotochromogenic, rapidly growing species isolated from a marine sponge. Int J Syst Bacteriol 37: 186–191.

[45] Izumi H, Gauthier ME, Degnan BM, Ng YK, Hewavitharana AK, et al (2010) Diversity of *Mycobacterium* species from marine sponges and their sensitivity to antagonism by sponge-derived rifamycin-synthesizing actinobacterium in the genus *Salinispora* . FEMS Microbiol Lett 313: 33–40. 20883497 10.1111/j.1574-6968.2010.02118.x

[46] Hirsch AM, Valdés M (2010) *Micromonospora*: An important microbe for biomedicine and potentially for biocontrol and biofuels. Soil Biol Biochem 42: 536–542.

[47] Lampert Y, Kelman D, Nitzan Y, Dubinsky Z, Behar A, et al (2008) Phylogenetic diversity of bacteria associated with the mucus of Red Sea corals. FEMS Microbiol Ecol 64: 187–198. 18355296 10.1111/j.1574-6941.2008.00458.x

