# Supplementary material for: Functional Gene-Guided Discovery of Type II Polyketides from Culturable Actinomycetes Associated with Soft Coral Scleronephthya sp
Source: PLoS One. 2012 Aug 7;7(8):e42847. doi: 10.1371/journal.pone.0042847 (PMC3413676; doi:10.1371/journal.pone.0042847)
Supplement: Table S1 — Media used for the isolation of actinomycetes from the soft coral Scleronephthya sp. (DOC) [file pone.0042847.s001.doc]

| Medium | Reference |
| --- | --- |
| M1 10 g soluble starch, 4 g yeast extract, 2 g peptone, 18 g agar, and 1 l of artificial seawater  M2 6 ml 100% glycerol, 1 g arginine, 1 g K_2_HPO_4_, 0.5 g MgSO_4_, 18 g agar, and 1 l of artificial seawater  M3 4 g yeast extract, 10 g malt extract, 4 g glucose, 18 g agar, and 1 l of artificial seawater  M4 0.1 g L-asparagine, 0.5 g K_2_HPO_4_, 0.001 g FeSO_4_, 0.1 g MgSO_4_, 2 g peptone, 4 g sodium propionate, 18 g agar, and 1 l of artificial seawater  M5 0.5 g yeast extract, 0.25 g tryptone, 0.75 g peptone, 0.5 g glucose, 0.5 g soluble starch, 0.3 g K_2_HPO_4_, 0.024 g MgSO_4_, 0.3 g sodium propionate, 18 g agar, and 1 l of artificial seawater  M6 10 g soluble starch, 1 g casein, 0.5 g K_2_HPO_4_, 18 g agar, and 1 l of artificial seawater | 7  7  43  10  12  43 |

**Supporting Information Legends**:

**Table S1** Composition of the six media for the isolation of actinomycetes from the soft coral *Scleronephthya* sp.
