# Supplementary material for: Functional Gene-Guided Discovery of Type II Polyketides from Culturable Actinomycetes Associated with Soft Coral Scleronephthya sp
Source: PLoS One. 2012 Aug 7;7(8):e42847. doi: 10.1371/journal.pone.0042847 (PMC3413676; doi:10.1371/journal.pone.0042847)
Supplement: Figure S1 — The diversity of actinomycetes recovered using six media. (DOC) [file pone.0042847.s002.doc]

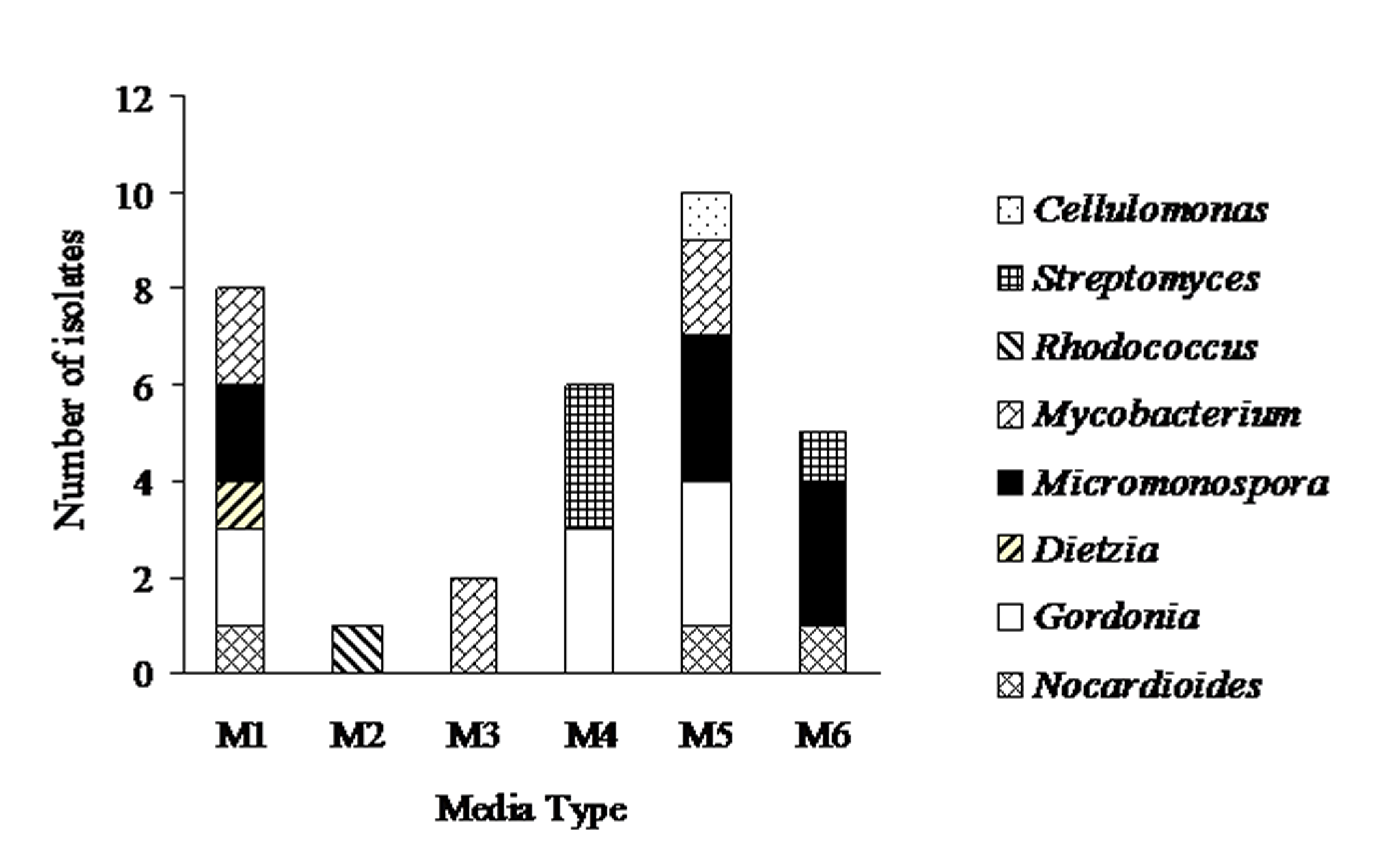


**Supporting Information Legends**:

**Figure S1** The diversity of actinomycetes recovered on the six media
